# Staging and Treatment Implications in Small Oral Squamous Cell Carcinoma with Bone Infiltration

**DOI:** 10.3390/biomedicines13030628

**Published:** 2025-03-05

**Authors:** Carolin Naegeli-Pullankavumkal, Raphael Ferrari, Thomas Gander, Martin Lanzer

**Affiliations:** Department of Oromaxillofacial Surgery, University Hospital Zürich, Rämistrasse 100, 8091 Zürich, Switzerland

**Keywords:** oral squamous cell carcinoma, staging, bone infiltration, prognosis, survival

## Abstract

**Background/Objectives:** Oral squamous cell carcinoma (OSCC) with bone infiltration is categorized as a T4 tumor regardless of its size. T4 tumors are an indication for postoperative radiotherapy, which could be overtreatment for small oral squamous cell carcinoma (SOSCC) with bone infiltration. **Methods:** A retrospective study of 189 patients with OSCC with the potential for mandibular infiltration was performed. The influence of the predictive variables on overall survival (OS) and disease-free survival (DFS) was assessed using the Kaplan–Meier method. A random forest approach was applied to determine the importance of each variable for survival in a multivariate context, and a partial correlation analysis was performed. **Results:** A statistical analysis of the effects of covariates suggested only a small influence of bone infiltration on OS. Patients with bone infiltration had a 5-year OS of 69%, and those without bone invasion had a 5-year OS of 71%. Age, lymph node metastasis, depth of invasion (DOI), and tumor size had the most decisive prognostic influence on survival. **Conclusions:** Bone infiltration appears to have less prognostic explanatory power than other known variables regarding OS. Therefore, adjuvant therapy should be carefully evaluated.

## 1. Introduction

Squamous cell carcinoma (SCC) accounts for approximately 90% of head and neck cancers (HNCs) [1,2]. It mainly originates from the epithelial cells of the oral cavity, pharynx, and larynx. Oral squamous cell carcinoma (OSCC) occurs in the wet mucosa of the lip, buccal mucosa, vestibule, alveolar process, retromolar area, tongue, floor of mouth, or hard palate. It may proliferate to the bone tissue of the maxilla or mandible. The literature describes the invasion of the mandibular bone with a prevalence of 12–56% [3,4,5].

However, the mechanisms underlying bone invasion in OSCC remain unclear. Two different patterns have been identified: infiltrative and erosive [6]. The infiltrative pattern follows tumor expansion, with little osteoclastic activity and the absence of an intervening layer of connective tissue. Tumor cells penetrate the cancellous space through the area of the resorbed alveolar ridge, incompletely healed extraction socket, or periodontal ligament [6,7]. The erosive pattern is characterized by osteoclast activity and a connective tissue layer along the tumor border. Mixed forms of erosive patterns that evolve into invasive growth have also been described [6,7,8].

Through the RANK-RANKL-OPG pathway, osteoclastogenesis in OSCC leads to the activation of osteoclasts and an imbalance in the activity of osteoclasts and osteoblasts [9,10]. The cytokines IL-1α, IL-6, TNF-α, and parathyroid hormone-related protein (PTHrP) promote the differentiation of osteoclasts and lead to bone invasion. This results in the release of growth factors that promote tumor growth [11].

Since the first TNM classification by the American Joint Committee on Cancer (AJCC) in 1977, OSCC with bone invasion has been categorized as T4 [12]. Tumors with invasion through the cortical bone into the medullary cavity are classified as T4a according to the AJCC Cancer Staging Manual (7th edition) published in 2010 [13,14]. However, tumor size itself is not considered in the classification of OSCC with bone infiltration. The TNM Classification of Malignant Tumours (8th edition) published in 2017 also includes the depth of invasion (DOI) and extranodal extension (ENE) in staging because these parameters have a significant impact on the outcome [12]. In a large meta-analysis with 40,808 patients, Almangush et al. [15] demonstrated good risk stratification through the adjustments in the 8th edition of the TNM tumor classification.

Prognosis-influencing factors can be related to the patient, the tumor itself, or the treatment. Patient-related factors, such as age, tobacco use, and high alcohol consumption, are well known [16,17]. Regarding tumor-related factors, the TNM classification allocates parameters that affect the outcome, such as the size of the primary tumor (T), regional lymph nodes (N), and metastasis (M). Recurrence and survival rates vary among tumor stages (I-IV) [13,18,19]. The importance of the DOI and ENE has been identified [20,21] and added to the TNM Classification of Malignant Tumours (8th edition). Histologic differentiation [22,23], perineural invasion [24,25,26], angiogenesis [27,28], human papillomavirus (HPV)-16 [29,30], and various other biomarkers are considered prognostic factors for OSCC. Treatment-related parameters and disease-free resection margins are clearly associated with higher survival rates and lower local recurrence [31,32,33]. The decision to administer adjuvant therapy in the treatment of OSCC is based on several factors, including pathological T and N staging after surgery of the primary tumor and its possible metastatic lymph nodes. Hence, the extent of tumor resection and lymphadenectomy contributes to patient morbidity and local and overall outcomes. Subsequent therapy in terms of radiotherapy (RT) alone or in combination with chemotherapy (RCT) in advanced-stage carcinoma also influences patient outcomes. The National Comprehensive Cancer Network (NCCN) Guidelines for Head and Neck Cancers, updated in March 2021, provide treatment recommendations [13,34,35,36].

Establishing an adequate treatment regimen demands a multidisciplinary evaluation and aims to maximize survival while preserving anatomical form and function. The extent of curative surgical treatment with the resection of OSCC and sufficient tumor-free margins is determined by tumor size, tumor location, and corresponding suspicious malignant lymph nodes. Marginal resection of the mandible is a surgical option when the malignoma approaches from the floor of the mouth or buccal cavity but is not attached to the mandible or periosteum. Segmental resection is indicated in cases of the infiltration of the cancellous mandible. Treatment concepts for the gross infiltration of adjacent soft tissue, the possible involvement of the periosteum with clinical fixation, or the radiological erosion of the cortical mandible have not been clearly defined [36,37,38,39]. Hence, bone involvement’s prognostic impact must be considered when planning surgical treatment.

According to the current guidelines, adjuvant RT is recommended for T4 tumors. However, this approach could be considered overtreatment in patients with SOSCC with bone infiltration in isolation without other adverse features, such as lymph node involvement, positive margins, perineural invasion, or lymphovascular invasion. In this study, we aimed to retrospectively investigate the prognostic impact of bone infiltration on the 5-year overall survival (OS) and 5-year disease-free survival (DFS) of patients with SOSCC at the Oromaxillofacial Department of the University Hospital Zürich and to evaluate the possible consequences for staging and treatment.

## 2. Materials and Methods

For this retrospective study, we identified patients with OSCC of the mandible or potentially mandible-infiltrating regions who received primary surgical treatment at the Oromaxillofacial Department of the University Hospital Zürich between January 2006 and December 2018 from the hospital’s database. We considered the alveolar crest of the mandible, lateral border of the tongue, floor of the mouth, and retromolar area as potential mandible-infiltrating regions. All the patients were treated with curative intent. Only the patients with a negative bone margin status of primary resection were included. The patients who received neoadjuvant treatment, those with non-resectable carcinoma, and those with inadequate information were excluded. We obtained permission to conduct this study from Swissethics (Swiss Ethics Committee on Research Involving Humans, BASEC-Nr. 2017-01065).

Clinicopathological data were obtained from medical records, including radiology, surgery, pathology, and tumor conference reports. The variables extracted for analysis included age, gender, tumor region, tumor size, bone infiltration, regional lymph node metastasis, perineural invasion (Pn), blood vessel invasion (V), lymphovascular invasion (L), surgical margin involvement, the minimum resection distance to the tumor, the DOI, adjuvant therapy, 5-year OS, and 5-year DFS. We investigated these variables in a subpopulation of patients with bone invasion. We considered T4a tumors up to 40 mm in size as SOSCC.

The patients were treated according to the corresponding up-to-date treatment guidelines [40,41]. A segmental mandibulectomy was indicated if bone infiltration was evident in presurgical staging. Adjuvant RT was indicated in advanced tumor categories (T3/T4), narrow or positive resection margins, perineural invasion, vascular invasion, and/or regional lymph node metastasis. Concomitant chemotherapy was indicated for patients with a higher histopathological risk of tumor recurrence, including those with resection margins of <5 mm or ENE.

We generated descriptive statistics for the sample and performed graphic analyses of 5-year OS and 5-year DFS using the Kaplan–Meier method and the corresponding survival curves. The log-rank test with a significance of *p* < 0.05 confirmed a significant difference between the survival curves of the various groups in the Kaplan–Meier analysis. We conducted a DFS analysis dependent on bone infiltration using the competing risk model when calculating the survival analysis; other causes of death were excluded. We investigated the influence of the explanatory variables age, gender, tumor region, tumor size, bone infiltration, regional lymph node metastasis, perineural invasion, blood vessel invasion, lymphovascular invasion, surgical margin involvement, the minimum resection distance to the tumor, the DOI, and adjuvant therapy on OS by using a random forest approach [42,43]. We used variable importance to identify important explanatory variables with respect to OS in the random forest fit [44]. Moreover, by constructing partial dependence plots for two selected variables (i.e., bone invasion and regional lymph node metastasis), we adjusted their explanatory power for the influence of the remaining variables [45] and then explored them in more detail. We performed all the statistical analyses using the statistical software R, URL https://www.R-project.org, accessed on 20 February 2020 [46], including the packages randomForestSRC R package version 2.9.1 [42], ggRandomForests R package version 2.0.1 [45], survival version 2.38 [47], and cr17 R package version 0.1.0 [48].

## 3. Results

Our study sample consisted of 189 patients: 118 men (62%) and 71 women (38%), with a mean age of 64 years and a median age of 63 years (range of 26–92 years). The location of the OSCC was mainly in the alveolar crest of the mandible (*n* = 71, 38%), followed by the lateral border of the tongue (*n* = 50, 26%), the floor of the mouth (*n* = 46, 24%), and the retromolar area (*n* = 22, 12%). Tumor size was graded according to the TNM classification, with T1 at <20 mm (*n* = 85, 46%), T2 at 21–40 mm (*n* = 70, 38%), and T3 at >40 mm (*n* = 29, 16%). A total of 130 patients (69%) showed no bone involvement, 7 (4%) presented erosion of cortical bone, and 52 (28%) showed infiltration into the spongiosa. Regional lymph node metastasis was negative in 112 patients (65%) and positive in 35 patients (20%), and ENE was present in 24 patients (14%). Adjuvant RT was administered to 39 patients (21%), and RCT was administered to 43 patients (23%); 107 patients (57%) had no additional therapy after surgery. The median follow-up duration was 60 months. Locoregional recurrence was diagnosed in 60 patients (32%) after a median follow-up period of 30 months. In the subpopulation that developed local recurrence, 15 patients (25%) were initially affected by bone infiltration. Bone infiltration was more frequent in patients with tumors >20 mm (T2, *n* = 29, 41%; T3, *n* = 20, 69%), lymph node metastasis (*n* = 12, 34%), ENE (*n* = 13, 54%), and DOI > 10 mm (*n* = 23, 66%).

A summary of the demographic and clinicopathological data of the study sample is shown in Table 1, along with the levels of measurement and distribution.

### 3.1. Descriptive and Graphical Analyses

In our study sample, bone involvement was present in 52 patients (27%) with infiltration, in 7 patients (4%) with erosion, and in 130 patients (69%) without any bone invasion. According to the TNM classification, we added the patients with erosion to the group with no bone involvement because further analysis of this subpopulation was not representative.

**Figure 1:**
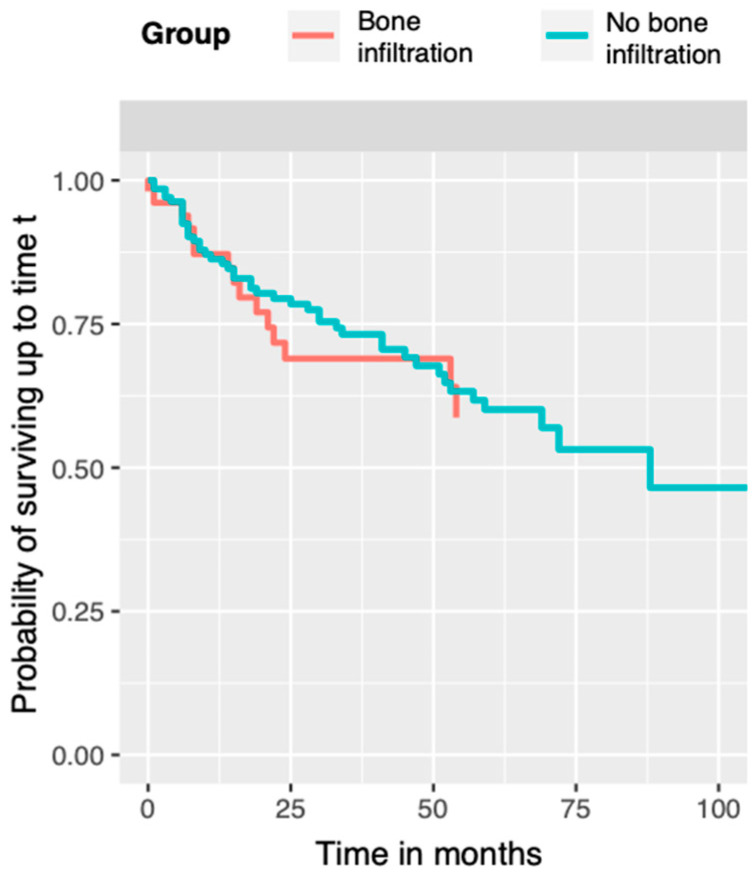
Disease-free survival (DFS) curves. Red represents the group with bone infiltration and blue represents the group without bone infiltration.

In the univariate Kaplan–Meier analysis, the patients with bone infiltration had a statistically significant lower 5-year OS, at 54% (95% confidence interval [CI] 41–72%), than the patients with no bone invasion (OS of 75%, 95% CI 67–84%; χ2 (1) = 9.5, *p* = 0.002). There was no significant difference in 5-year DFS between the patients with and without bone infiltration. In the patients with bone infiltration, DFS was 59% (95% CI 43–80%), whereas in the patients without bone invasion, DFS was 60% (95% CI 51–71%) (Figure 1).

**Figure 2:**
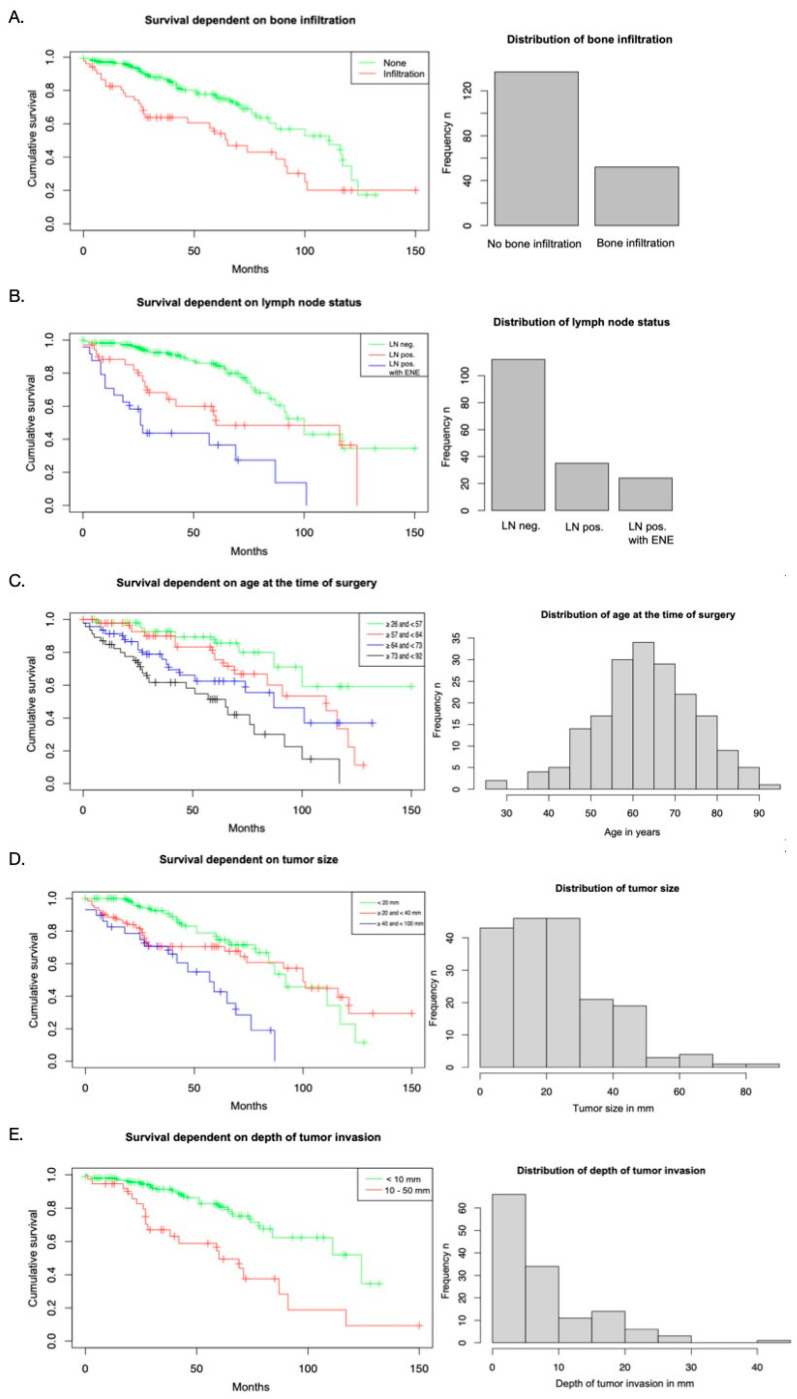
Univariate analysis of survival. The Kaplan–Meier curves present survival depending on (**A**) bone infiltration, (**B**) lymph node status, (**C**) age at the time of surgery, (**D**) tumor size, and (**E**) depth of tumor invasion.

Lymph node metastasis, age, tumor size, and the DOI had prognostic power for 5-year OS. The patients with ENE had a lower OS (37%, 95% CI 20–66%) than those with positive lymph node metastasis (49%, 95% CI 32–74%) or negative lymph node metastasis (84%, 95% CI 76–93%; χ2 (2) = 33.9, *p* < 0.001) (Figure 2).

The age distribution of the study sample is presented as a histogram in Figure 2. When comparing the 5-year OS rates of the four age groups, it was found that OS consistently decreased as age increased. The 5-year OS rate was 86% (95% CI 75–99%) for the group ranging from 26 to 57 years, 76% (95% CI 62–92%) for the group ranging from 57 to 64 years, 62% (95% CI 48–81%) for the group ranging from 64 to 73 years, and 51% (95% CI 37–71%) for the group ranging from 73 to 92 years (χ2 (3) = 22.1, *p* < 0.001) (Figure 2).

The patients with tumors < 20 mm had a substantially higher 5-year OS (75%, 95% CI 64–87%) than those with tumors > 40 mm (43%, 95% CI 26–72%; χ2 (2) = 15.5, *p* < 0.001). The patients with DOI ≤ 10 mm had a higher 5-year OS (81%, 95% CI 71–91%) than those with DOI > 10 mm (49%, 95% CI 34–73%; χ2 (1) = 13.5, *p* < 0.001) (Figure 2).

### 3.2. Random Forest Analysis of OS

**Figure 3:**
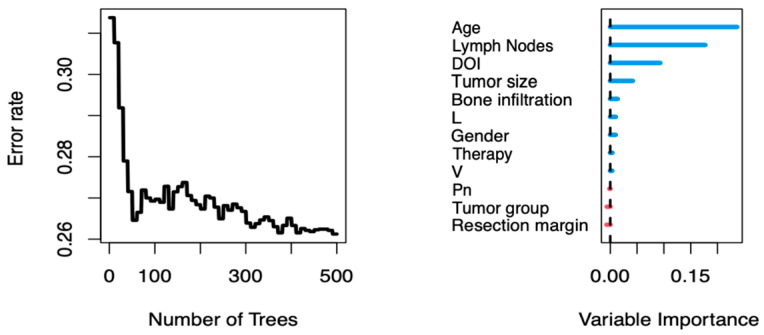
The out-of-bag error rate for the entire random forest fit and variable importance for individual explanatory variables based on permuted out-of-bag cases.

We computed the random forest fit using 12 potential explanatory variables. Figure 3 shows the out-of-bag error (1–C, where C is Harrell’s concordance index) of the resulting fit. An out-of-bag error of <0.5 indicated that the model fit had some predictive power, while a value of 0 indicated a perfect prediction. In our case, the error rate stabilized at approximately 0.26, indicating that the random forest fit, and thus the underlying dataset, explained a fair proportion of the variability in OS. Next, we investigated the importance of each explanatory variable using permuted out-of-bag cases. We found that age and regional lymph node metastasis seemed to have the most significant impact on 5-year OS, followed by the DOI, tumor size, bone infiltration, lymphovascular invasion, gender, adjuvant therapy, blood vessel invasion, perineural invasion, tumor region, and surgical margin involvement, all with far less explanatory power (Figure 3).

### 3.3. Partial Variable Dependence Concerning Bone Infiltration

**Figure 4:**
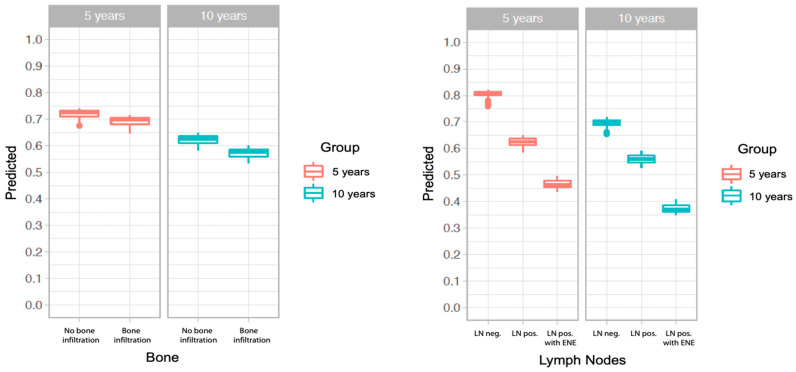
Overall survival (OS) in view of A. bone infiltration and B. lymph node status, adjusted for the remaining covariates. Red represents the 5-year-OS and blue represents the 10-year-OS.

The partial variable dependence plot displays the estimated OS at predefined time points by varying a single factor adjusted for the influence of the remaining variables. A comparison of the plots of bone infiltration and regional lymph node metastasis revealed a much smaller impact of bone infiltration on OS (Figure 4). The model predicted a markedly decreased 5-year OS in the patients with regional lymph node metastasis (~63%) or ENE (~46%) compared with the patients without regional lymph node metastasis (~81%). Conversely, bone infiltration seemed to play less of a role in 5-year OS, which was approximately 69% for the patients with bone infiltration and approximately 71% for the patients without bone invasion.

## 4. Discussion

This retrospective study aimed to evaluate the prognostic value of bone infiltration in SOSCC patients. Ebrahimi et al. [48] identified medullary invasion as an independent predictor of reduced OS. In addition, they found that tumor size regulated survival. They suggested staging tumors from T1 to T3 according to their size and upgrading to one T stage in cases of bone infiltration.

Controversial results have been published regarding the role of bone invasion in the outcome of OSCC [49,50,51,52,53]. In the AJCC Cancer Staging Manual (7th edition), bone involvement is defined as infiltration into the medullary cavity, not only the erosion of the cortical bone. Some studies have investigated the prognostic impact of bone invasion, particularly in small tumors. Fives et al. [51] showed significantly worse local control (LC) and OS in patients with tumors <40 mm in size with bone invasion. Okura et al. [54] investigated the impact of different types of bone invasion and differentiated between the mandibular canal and medullary invasion. In contrast to mandibular canal invasion, medullary invasion was not an independent predictor of OS but was an independent factor for distant control. Kuk et al. [55] showed that bone invasion was an independent prognostic factor for DFS in patients with OSCC. However, in patients with SOSCC < 40 mm, bone involvement did not significantly affect disease progression in their study group. Fried et al. [52] came to similar conclusions.

Based on our analysis, we propose that DFS is less influenced by bone infiltration than is generally assumed. We found a similar probability of tumor recurrence with and without bone infiltration (Figure 1). Considering that most of our patients (84%) had tumors < 40 mm, these results are consistent with those of other studies [53,56,57,58]. Patel et al. [59] did not identify any difference in local control or survival in patients with either cortical invasion alone or medullary cavity invasion.

As mentioned previously, bone infiltration can occur in erosive, infiltrative, or mixed patterns. Our pathological reports did not differentiate between bone invasion patterns. Therefore, further analysis was not possible. The histological pattern correlates with the clinical behavior of OSCC. While erosive types present a less aggressive formation, infiltrative tumors contribute to higher primary, regional, and distant recurrences. Wong et al. [60] determined a 3-year DFS of 30% for the infiltrative pattern and 73% for the erosive pattern. Furthermore, there was no correlation between the infiltrative pattern and overall tumor size. We hypothesize that these histological patterns and their clinical behavior explain the controversial results of earlier studies as well as ours [55,60,61].

Because bone invasion did not play an essential role in our cohort, we evaluated the prognostic impact of additional factors on the survival of patients with OSCC. We found that lymph node metastasis, age, tumor size, and DOI contributed to a lower OS. Positive lymph node metastasis and ENE lead to poor outcomes [62,63]. Shaw et al. [64] revealed the importance of ENE in cervical lymph nodes as an adverse prognostic factor in OSCC. Local recurrence, distant metastases, and regional failure increased in the presence of ENE; 5-year OS was 31% for microscopic ENE and even 19% for macroscopic ENE. Our univariate and multivariate analyses support this hypothesis. Therefore, precise staging, an adequate surgical approach, and adjuvant therapy for lymph node metastasis are crucial.

Age was identified as an essential prognostic variable in the analyses. Consistent with our results, several studies have described a decrease in survival as age increases [65,66]. Age has been a controversial prognostic parameter [16,67]. It is widely recognized that with age, the chances of developing carcinoma increase because of the complex biological processes associated with aging. More prolonged exposure to environmental carcinogens, the accumulation of DNA damage, mutations over time, and the disruption of DNA repair and cell growth regulation systems can transform normal cells into carcinoma [68,69]. In terms of survival, Leonicini et al. [70] found age, among other factors, to be an adverse variable for OS in head and neck cancer. In contrast, Wang et al. [23] did not find age to be a prognostic parameter for recurrence. Age itself may not be a prognostic variable; however, it can be considered a negative prognostic indicator because more comorbidities occur with advanced age. Therefore, age should be acknowledged as biological age rather than chronological age.

In the TNM staging system, tumor dimension (T1–4), regional lymph node involvement (N0–3), and the presence of distant metastases (M0–1) are incorporated to form stages I-IV, which are used for prognosis, treatment decisions, and comparisons of the treatment results. Thus, tumor size has been critical in staging, prognosis, and therapy for decades. In this study, tumor size had some prognostic properties. Multiple studies have investigated this correlation and detected a relationship between tumor size and survival probability. Patients with small early-stage tumors present a 5-year OS of 80–90%; however, 5-year OS markedly decreases to 40% in advanced-stage tumors [71,72,73].

A recent change in the TNM classification is the addition of the DOI to the T category. Tan et al. [74] determined the prognostic significance of the DOI in oral tongue cancer and divided their study population into group A (DOI < 4 mm) and group B (DOI ≥ 4 mm). They reported an adverse effect of the DOI on survival: 5-year OS was 68.8% in group A and 41.6% in group B, DFS was 67.1% in group A and 41.1% in group B, and local recurrence-free survival was 89.5% in group A and 65.4% in group B. Lee et al. [75] analyzed patients from the National Cancer Database and ascertained the 5-year OS of tumors staged using the AJCC Cancer Staging Manual (7th edition). A higher DOI was associated with a lower 5-year OS. Our investigation supports these results, as the DOI significantly impacted prognosis. Pollaers et al. [76] examined the impact of the DOI on survival by comparing the AJCC Cancer Staging Manual 7th and 8th editions. They concluded that OS, grouped by stage according to the two editions, varied significantly among the cancer stages. Furthermore, the T stage of the AJCC Cancer Staging Manual (8th edition) showed a more accurate relation to DFS than the T stage of the AJCC Cancer Staging Manual (7th edition). Considering DOI as complementary information allows for a more precise T-classification and, consequently, accurate therapy.

With controversial results regarding the influence of bone invasion and multiple variables influencing patient outcomes, we conducted a random forest analysis to determine the highest prognostic impact of the collected data between the potentially correlating variables. Age and lymph node metastasis were identified as the leading predictive variables of OS, whereas bone infiltration played only a minor role among the other prognostic factors. Bone invasion was potentially correlated with lymph node metastasis, age, DOI, and tumor size. According to the Kaplan–Meier analysis, bone infiltration significantly impacted survival. We hypothesized that this could be explained by the increased multimorbidity and associated lower survival in the group with bone infiltration. However, when comparing OS (Figure 4) with respect to bone infiltration and regional lymph node status, each controlled for the other identified factors, it was evident that the absence of bone involvement did not improve the outcome as significantly as did the absence of lymph node metastasis. Hence, the question arises as to whether upstaging tumors to T4 in cases of bone infiltration is justified. Many studies, including the present one, have demonstrated a limited contribution of mandibular invasion to OS in patients with OSCC [52,54,55,77].

Multiple variables influence the course of the disease and a patient’s life. It is difficult to draw a clear picture because all the variables must be viewed in an overall context. A retrospective study may have the most sources of error arising from confounding factors, selection biases, and patient biases, e.g., missing data and comorbidities. In addition to sufficient population size and more variables, such as the histological pattern of bone invasion, a control group for evaluating the impact of adjuvant therapy is necessary, which is ethically and practically unfeasible in this setting. Therefore, our study has limitations in the interpretation of the results.

## 5. Conclusions

We reviewed multiple prognostic factors and the role of bone infiltration in SOSCC. Although a univariate analysis revealed that the latter might be a predictive factor for survival, a multivariate analysis showed far less of an impact on survival. Lymph node metastasis, age, DOI, and tumor size influenced OS in our study sample to a greater extent than bone invasion. When staging OSCC according to the TNM classification, the variables of age with comorbidities and bone invasion with histological patterns should also be considered. The decision for adjuvant therapy requires consideration of the patient’s overall situation.

However, further investigations with an appropriate study design are needed to evaluate the role of bone infiltration in SOSCC in isolation of other adverse features. The decision to administer adjuvant therapy should be evaluated carefully and individually to provide patients with maximum safety and comfort.

## Figures and Tables

**Table 1:** Demographic and clinicopathological data.

	All Patients	Bone Invasion Yes/No	Classification/Subdivision
Total No of Patients	189	52/137 (28%)	
Age	Mean 64		Four equally sized groups: “[26,57)”, “[57,64)”, “[64,73)” and “[73,92)”
	Median 63		
Gender			Two groups: “Men” and “Women”
Men	118 (62%)	35/83 (30%)	
Women	71 (38%)	17/54 (24%)	
Subsite of Primary Lesion			Four groups: “Alveolar Crest of Mandible”, “Lateral Border of Tongue”, “Floor of Mouth”, and “Retromolar Area”
Alveolar Crest of Mandible	71 (38%)	43/28 (61%)	
Lateral Border of Tongue	50 (26%)	0/50 (0%)	
Floor of Mouth	46 (24%)	9/37 (20%)	
Retromolar Area	22 (12%)	0/22 (0%)	
Tumor Size			Three groups: “[−0.1,20)”, “[20,40)”, and “[40,100)”
<20 mm	85 (46%)	3/82 (4%)	
20–40 mm	70 (38%)	29/41 (41%)	
>40 mm	29 (16%)	20/9 (69%)	
Bone Invasion			Two groups: “None” and “Infiltration”
None	130 (69%)		
Erosion	7 (4%)		
Infiltration	52 (27%)		
Lymph Node Invasion			Three groups: “None”, “Metastasis”, and “ENE”
None	112 (66%)	27/85 (24%)	
Metastasis	35 (20%)	12/23 (34%)	
ENE	24 (14%)	13/11 (54%)	
Perineural Invasion	39 (26%)	17/22 Pn1 (44%) 23/86 Pn0 (21%)	Two groups: “No” and “Yes”
Lymphatic Invasion	15 (10%)	11/4 L1 (73%) 26/102 L0 (20%)	Two groups: “No” and “Yes”
Vessel Invasion	6 (4%)	4/2 V1 (67%) 33/106 V0 (24%)	Two groups: “No” and “Yes”
Surgical Resection Margins			Three groups: “Free”, “Close”, and “Involved”
Free	172 (91%)	42/130 (24%)	
Close < 5 mm	100 (65%)	29/71 (29%)	
Involved	16 (9%)	10/6 (63%)	
Depth of Invasion			Two groups: “[−0.1,10)” and “[10,50)”
≤5 mm	66 (35%)	2/64 (3%)	
>5 and ≤10 mm	34 (18%)	4/30 (12%)	
>10 mm	35 (18%)	23/12 (66%)	
Unknown	54 (29%)	23/31 (43%)	
Adjuvant Therapy			Three groups: “None”, “Radiotherapy”, and “Radiochemotherapy”
None	107 (56%)	7/100 (7%)	
Radiotherapy	39 (21%)	20/19 (51%)	
Radiochemotherapy	43 (23%)	25/18 (58%)	
Tumor Recurrence	60 (32%)	15/45 (25%)	Two groups: “No” and “Yes”
Interval to Recurrence	Mean 37 months		
No of Deceased	67 (35%)	29/38 (43%)	Two groups: “No” and “Yes”
Interval to Death	Mean 49 months		

Table 1 Overview of the assessed variables in the sample population. The column entitled “Classification/Subdivision” shows how the respective variables were used in the statistical assessments.

## Data Availability

The data that support the findings of this study are available from the corresponding author, C.N.-P., upon request. The data are not publicly available due to an ongoing study.
